# Influence of arboreal components on the physical-chemical characteristics of the soil under four silvopastoral systems in northeastern Peru

**DOI:** 10.1016/j.heliyon.2021.e07725

**Published:** 2021-08-08

**Authors:** Héctor V. Vásquez, Leandro Valqui, Leidy G. Bobadilla, Carlos I. Arbizu, Julio C. Alegre, Jorge L. Maicelo

**Affiliations:** a Dirección de Desarrollo Tecnológico Agrario, Instituto Nacional de Innovación Agraria (INIA), Av. La Molina 1981, Lima 15024, Peru; b Universidad Nacional Agraria la Molina (UNALM), Av. La Molina s/n, Lima 15012, Peru

**Keywords:** Organic matter, Mechanical resistance, Treeless system, Density, Soil carbon

## Abstract

Silvopastoral systems (SPS) are presented as an alternative for the protection and recovery of soils; however, the relationship between the tree component and the physical-chemical characteristics of the soil is unknown. Thus, the objective of this study was to evaluate the physical-chemical characteristics of the soil under four silvopastoral systems (SPS), alder (*Alnus acuminata*), pine (*Pinus patula*), cypress (*Cupressus macrocarpa*), and pona (*Ceroxylon quindiuense*), and a treeless system (TS) in the Amazonas region. A completely randomized design (CRD) with five treatments and three replicates was used. The experimental units were sampled at two depths, 0–15 and 15–30 cm. The parameters evaluated were pH, electrical conductivity (dS/m), organic matter (%), phosphorus (ppm), potassium (ppm), cation exchange capacity (meq/100 g), porosity (%), mechanical resistance (kg/cm^2^), bulk density (gr/cm^3^), moisture (%) and total carbon (t/ha). The results were analyzed by analysis of variance (α = 0.05 %) and Tukey's test of means (p ≤ 0.05). The systems presented strong acidic pH values (4.11–5.61), which resulted in high organic matter contents in all systems (6.74–9.99 %). The highest phosphorus content was in the SPS with alder (12.64 ppm), and the highest potassium content was in the SPS with cypress (382.33 ppm). Porosity in all systems was higher than 60 %. The highest bulk density was between 15 and 30 cm, and the highest percentage of moisture was in the surface layer (0–15 cm). The mechanical strength was higher in the SPS with cypress (2.62 kg/cm^2^). For all the systems evaluated, the highest carbon stock was found in the first 15 cm. The SPS with pine had the best soil characteristics and carbon sequestration (149.05 t/ha).

## 1 Introduction

In Latin America, one of the main factors causing land use changes is extensive livestock farming due to increasing deforestation when establishing new pastures (McGroddy et al., 2015). This scenario has raised the need to implement various actions that can promote the development of sustainable livestock farming, such as an alternative to encourage the natural regeneration of vegetation and the conservation of forests in the installation of silvopastoral systems (Arciniegas-Torres and Flórez-Delgado, 2018).

Peru is one of 17 countries that represent 70 % of the planet's total biodiversity and has great forestry potential, as it is home to 13 % of the Amazon rainforests, hosting 72 million hectares of forests. The total carbon stock in the biomass in this area could be up to 10.9 billion tonnes of carbon (Ministry of Environment, 2014; Food and Agriculture Organization, 2016). However, these resources are not well managed, and high rates of deforestation are recorded, causing soil degradation that affects crop production and environmental sustainability (Rojas et al., 2019). Deforestation resulting from activities such as land use changes, forestry, and agriculture is responsible for 60 % of Peru's greenhouse gas emissions. During the 1990–2015 period, the deforested area was 12 399 577.08 ha, which represents 12 % of the Amazon forest area, and the Amazon region has a deforested area of 1 010 590.75 ha (Ministry of Environment, 2015).

Livestock farming in the Amazonas region is highly debated because it is associated with deforestation, low productivity and high rates of soil degradation, which affects the sustainability of production areas (Institute of Investigations of the Peruvian Amazon, 2004). It is argued that to mitigate climate change, it is necessary to promote and develop sustainable livestock systems that allow the revaluation and recovery of degraded areas. Silvopastoral systems are one option available to reverse the degradation processes of primary forests and grasslands by increasing the physical protection of the soil and contributing to the recovery of fertility with nitrogen-fixing species and trees with taproots that take advantage of deep layers and recycle soil nutrients (Alonso, 2011).

De Souza et al. (2021) showed that after four years of installing silvopastoral systems in Brazil, the soil carbon and nitrogen pools increased compared to regenerated soil. Polanía-Hincapié et al. (2021) demonstrated that the use of silvopastoral systems in Colombia contributed to the recovery of the physical characteristics of the soil, such as bulk density and penetration resistance, which could be associated with the increased carbon content of the soil, as it is assumed that a higher biomass, above and below ground, will be produced.

In Peru, silvopastoral systems have not yet been implemented on a massive scale because their benefits are unknown, so the objective of this research is to determine the influence of the arboreal components of four silvopastoral systems associated with alder (*Alnus acuminata*), cypress (*Cupressus macrocarpa*), pine (*Pinus patula*), and pona (*Ceroxylon quindiuense*) on the physical-chemical characteristics of the soil in northeastern Peru.

## 2 Materials and methods

### 2.1 Study area

**Figure 1:**
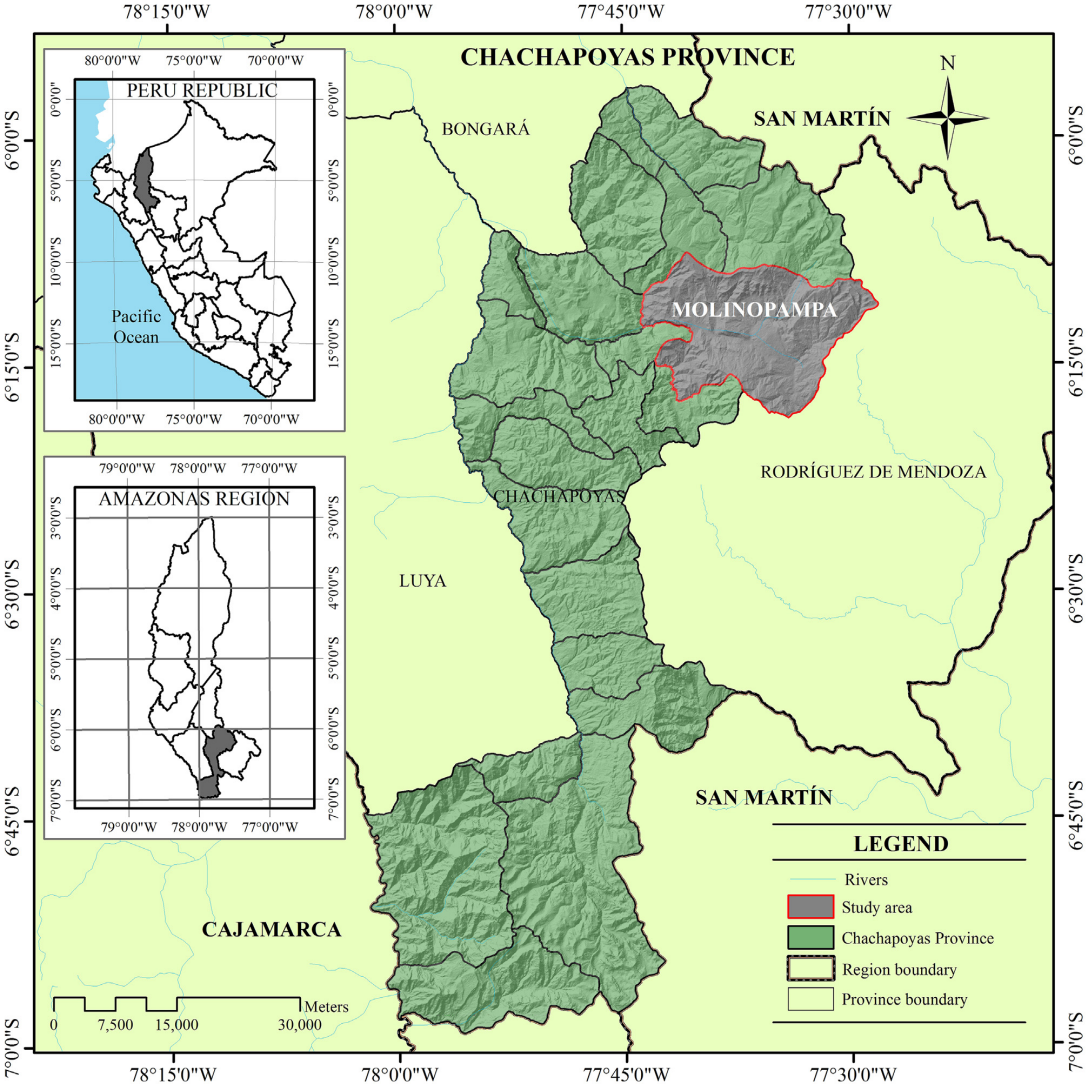
Study area.

The present study was carried out in the district of Molinopampa, Amazonas region, Peru (Figure 1). It is located at an altitude of 2 421 m above sea level and has an average minimum temperature of 10 °C, a maximum temperature of 26 °C, annual rainfall of 1 250 mm and relative humidity of 82 % (Servicio Nacional de Meteorología e Hidrología del Perú [SENAMHI], 2019).

This area has a very humid and cold temperate climate, where the months with highest rainfall are from November to March, the predominant land uses are livestock and the borders of protected lands, and the lithology of the soil corresponds to formations of the Goyllarisquizga Group and Chonta Formation (Regional Government of Amazonas, 2010).

The district of Molinopampa has a variety of terrains with different characteristics according to each ecological level; those located in flat areas are alluvial terraces with deep soils of a loamy to sandy texture and a high organic matter content due to their acidity. The areas that have hills with slight slopes are medium deep soils of a loamy to clayish texture, with a slightly acidic pH and medium fertility. Finally, the highest areas are characterized by slightly sloping terrain with deep soils, a high organic matter content, and a slightly acidic pH; in the study area, the clayish loamy textural class predominates (Oliva, 2016).

In the study area, silvopastoral systems predominate with forest species such as pine, alder, cypress, pona and open field systems, and the main forage species include rye grass (*Lolium multiflorum*) and clover (*Trifolium repens),* representing 62.7 % (López, 2019). Fifty-seven percent of producers are mainly engaged in livestock farming, of which 18 % manage silvopastoral systems and 67 % have more than six hectares as an SPS (Vásquez et al., 2020).

### 2.2 Silvopastoral systems evaluated

Four silvopastoral systems and one treeless system were evaluated in the Molinopampa cattle basin. These systems have the following characteristics:

The SPS with alder (*Alnus acuminata*) consisted of trees located in strips (seven years old and 10 m in height); the SPS with pine (*Pinus patula*) consisted of trees located in strips (12 years old and 12 m in height); the SPS with cypress (*Cupressus macrocarpa*) consisted of trees located in the form of a living fence (12 years old and 14 m high); the SPS with pona (*Ceroxylon quindiuense*) consisted of scattered trees in the paddock (70 years old and 23 m high); and the system without trees (TS) consisted only of grassland.

The floristic composition of the four silvopastoral systems contained a higher percentage by grasses such as *Dactylis glomerata* L. and *Lolium multiflorum*, followed by weeds such as *Cyperus* sp., *Paspalidium geminatum,* and *Sporobolus indicus* and legumes such as *Trifolium repens*.

### 2.3 Study design and evaluated parameters

A completely randomized design (CRD) with five treatments (four SPS and one TS) and three replicates was used. The experimental units (cattle herds) had an average density of 245 trees. Soil sampling was performed in each of the experimental units following the methodology of Mendoza and Espinoza (2017), with 18 m^2^ plots located under the trees for the SPS and in the open field for the TS. We used a 50 × 50 cm trial pit and evaluated two depths: 0–15 and 15–30 cm. The samples were analyzed at the soil laboratory of the National Agrarian University of La Molina, and the following parameters were recorded (Table 1):

**Table 1:** Methods carried out for the parameters evaluated.

Soil property	Method of determination
**Chemical parameter**	
Hydrogen potential (pH)	Potentiometer method
Electrical conductivity (CE) (dS/m)	Reading of aqueous extract at a 1:1 soil to water ratio
Organic matter (OM)	Walkley and Black's method
Available phosphorus (P) (ppm)	Modified Olsen method; 0.5 M NaHCO_3_ extract, pH 8.5
Potassium (K) (ppm)	Ammonium acetate extraction method
Cation exchange capacity (CEC) (meq/100 g)	Saturation with ammonium acetate (CH_3_–COOOCH_4_) N; pH 7.0

**Physical parameters**	
Porosity (%)	(1-Da/2.65) ∗100
Bulk density (g/cm^3^)	Soil extraction with cylinders of known volume. Oven dried at 105 °C for 24 h
Mechanical resistance (kg/cm^2^)	Penetrometer
Moisture content (%)	Gravimetric

Source: Bazán (2017).

Carbon was determined by applying the equations proposed by Arévalo et al. (2013):

(1) PVs (t/ha) = DA∗Ps∗10 000

where PVs = the weight of the soil, DA = bulk density (kg/m^3^), Ps = depth (cm) and 10 000 = a constant.

(2) CS (t/ha) = (PVs ∗ %C)/100

where CS = soil carbon, PVs = the weight of the soil; % CO = percentage of organic carbon, and 100 = a conversion factor.

### 2.4 Statistical processing and analysis

The results were subjected to an analysis of variance, and differences were compared using Tukey's test at 5 % significance. The analysis was performed using R software version 3.6.0.

## 3 Results and discussion

### 3.1 Chemical characteristics of the soils

#### 3.1.1 Hydrogen potential

This variable showed significant differences in the different systems (p = 0.0275). Table 2 shows that soil pH varied across the systems evaluated, presenting strongly acidic values in the SPS with pine (4.37 ± 0.32) and SPS with pona (4.09 ± 0.20). In addition, as the sampling depth increased, the pH levels increased from 4.75 ± 0.84 to 4.99 ± 0.99. Akinde et al. (2020) observed similar pH values for continuously cultivated soils in Nigeria, as they recorded pH values of 4.71 at a depth of 0–15 cm and 4.87 at a depth of 15–30 cm. On the other hand, the results of the present study were similar to those reported in Colombia from farms planted with *Brachiaria humidicola* associated with 3 tree species: *Anadenanthera peregrina, Pithecellobium guachapele* and *Acacia mangium*, recording acidic pH values ranging from 4.7 to 5.2 (Páez et al., 2014). In Costa Rica, Rojas et al. (2009) recorded pH values above 6.7 in six silvopastoral systems based on the combination of *Brachiaria brizantha* and *Hyparrhenia rufa* with native timber species in the dry tropics.

**Table 2:** Analysis of the effects of system type (S) and depth (D) on soil chemical characteristics.

System (S)	Chemical characteristics of the soils					
	pH (1:1)	CE (dS/m)	Organic matter (%)	Phosphorus (ppm)	Potassium (ppm)	CCI (meq/100 g)
SPS with alder	5.30 ± 0.73 a	0.22 ± 0.12 a	6.74 ± 5.23 a	12.68 ± 13.54 a	254.50 ± 127.98 ab	32.93 ± 4.50 a
SPS with pine	4.37 ± 0.32 a	0.08 ± 0.05 a	9.99 ± 7.63 a	2.97 ± 1.03 a	122.83 ± 91.28 b	30.51 ± 11.86 a
SPS with cypress	5.61 ± 1.26 a	0.28 ± 0.24 a	7.81 ± 5.73 a	12.64 ± 9.37 a	382.33 ± 150.88 a	34.40 ± 9.96 a
SPS with pona	4.11 ± 0.19 a	0.11 ± 0.06 a	8.11 ± 6.11 a	2.65 ± 1.64 a	227.33 ± 103.63 ab	31.49 ± 6.43 a
TS	4.99 ± 0.79 a	0.18 ± 0.13 a	9.17 ± 5.51 a	4.99 ± 3.15 a	218.42 ± 66.70 ab	35.72 ± 5.55 a

**Depth (D)**						
0–15 cm	4.76 ± 0.83 a	0.25 ± 0.17 a	13.38 ± 3.57 a	7.85 ± 8.61 a	293.68 ± 143.82 a	36.96 ± 7.26 a
15–30 cm	4.99 ± 0.99 a	0.09 ± 0.07 b	3.34 ± 1.57 b	6.52 ± 8.42 a	188.48 ± 103.39 b	29.06 ± 6.33 b

**Interaction (S × D) (cm)**						
SPS with alder 0 – 15	5.22 ± 0.52 a	0.31 ± 0.11 ab	10.96 ± 3.80 a	12.23 ± 12.18 a	282.67 ± 154.62 ab	35.57 ± 4.25 a
SPS with alder 15 – 30	5.37 ± 1.03 a	0.12 ± 0.03 ab	2.52 ± 0.82 b	13.13 ± 17.59 a	226.33 ± 121.08 ab	30.29 ± 3.42 a
SPS with pine 0 – 15	4.27 ± 0.43 a	0.13 ± 0.03 ab	16.07 ± 5.08 a	2.83 ± 0.91 a	174.67 ± 112.45 ab	37.87 ± 13.03 a
SPS with pine 15 – 30	4.47 ± 0.21 a	0.04 ± 0.01 b	3.90 ± 2.95 b	3.10 ± 1.32 a	71.00 ± 11.14 b	23.15 ± 4.37 a
SPS with cypress 0 – 15	5.34 ± 1.27 a	0.40 ± 0.31 a	12.67 ± 3.06 a	15.35 ± 12.60 a	453.67 ± 171.00 a	37.33 ± 11.68 a
SPS with cypress 15 – 30	5.87 ± 1.47 a	0.16 ± 0.12 ab	2.95 ± 1.39 b	9.93 ± 6.24 a	311.00 ± 111.37 ab	31.47 ± 9.25 a
SPS with pona 0 – 15	4.04 ± 0.21 a	0.15 ± 0.04 ab	13.25 ± 3.70 a	2.87 ± 2.11 a	294.00 ± 113.86 ab	35.20 ± 1.88 a
SPS with pona 15-30	4.17 ± 0.19 a	0.06 ± 0.02 ab	2.97 ± 0.75 b	2.43 ± 1.46 a	160.67 ± 23.50 ab	27.79 ± 7.65 a
TS 0-15	4.92 ± 0.86 a	0.26 ± 0.12 ab	13.96 ± 2.25 a	5.96 ± 3.57 a	263.42 ± 69.52 ab	38.84 ± 5.18 a
TS 15-30	5.06 ± 0.90 a	0.09 ± 0.05 ab	4.37 ± 1.34 b	4.02 ± 3.05 a	173.42 ± 14.60 ab	32.61 ± 4.58 a

pH: Hydrogen potential, EC: Electrical conductivity and CCI: Capacity of cationic interchange, ppm: parts per million, meq/100 g: milliequivalents per 100 grams, dS/m: Decisiemens per meter, cm: centimeter, (a, b) Different letters vertically indicate significant differences.

*Pinus patula* trees tend to acidify the soils of the silvopastoral system and help to improve soil organic matter levels. In addition to their arrangement in strips, trees in SPS contribute to the availability of nutrients, while the pH is influenced by the presence of rainfall (Di et al., 2018; Osorio, 2012). On the other hand, as organic matter levels increase, pH decreases (Cruz-Macías et al., 2020).

#### 3.1.2 Electrical conductivity

Regarding this variable, there were highly significant differences for depth (p = 0.0019); the EC values decreased as soil depth increased from 0.25 ± 0.17 dS/m (0–15 cm) to 0.09 ± 0.07 dS/m (15–30 cm). This trend differed from that obtained by Cantú et al. (2018), who found an increasing trend of electrical conductivity values with increasing depth for a grassland area in the silt loam soils of northeastern Mexico. This difference could be because conductivity values will fluctuate more at higher silt concentrations (Vásquez et al., 2014).

#### 3.1.3 Organic matter

The OM levels were high in all systems, with an average value of 6.74 ± 5.23 % (Table 2). These values are higher than those reported in an SPS with pona (6.63 % OM) in Pomacochas, Amazonas, by Maicelo (2012). The OM levels showed highly significant differences across depths (p = 0.0000). These values decreased as soil depth increased and were similar to results found by De Souza et al. (2021), who demonstrated that the tree component had a positive influence on organic matter accumulation when compared to a system without trees.

The highest percentage of organic matter was obtained for the SPS with pine (9.99 ± 7.63 %); this value was higher than that obtained by Oliva et al. (2017), who reported a value of 5.68 % OM in an SPS with pine. The difference in these results could be due to the difference in the sampling seasons, since the highest values were recorded in wet seasons (De Souza et al., 2021), which is the season in which the samples from the present study were taken.

#### 3.1.4 Available phosphorus

The system that presented the highest P value was the SPS with alder (12.68 ± 13.54 ppm), Saucedo (2018) also reported a higher value with a silvopastoral system with alder distributed in alleys (9.68 ± 6.71 ppm) in Molinopampa, Peru. Alder in silvopastoral systems contributes to the improvement of phosphorus contents, as mentioned by Navia et al. (2001), who proved that phosphorus increases after establishing a silvopastoral arrangement with alder. These results were similar to those reported by Akinde et al. (2020), who evaluated the phosphorus content for six types of agricultural land uses in Nigeria. These results may also be due to the decrease in organic matter and organic phosphates, since the residues of leaves and decomposing material are found in the surface layer of the soil (Bailon, 2018).

#### 3.1.5 Potassium

For the potassium (K) content, a highly significant difference was found between systems (p = 0.0079) and depth (p = 0.0130), with a low level found for the SPS with pine (122.83 ± 91.28 ppm) and a high value for the SPS with cypress (382.33 ± 150. 88 ppm). These results are higher than those reported by Crespo (2008) in Cuba, who obtained 199 ppm for a silvopastoral system of *Leucanena* plus grass pasture with an establishment of 15 years. Likewise, Oliva et al. (2016) reported values of 109.95 ppm for an SPS with alder, which was lower than those obtained in this research.

### 3.2 Physical characteristics of the soils

#### 3.2.1 Mechanical resistance

For the mechanical resistance, the results show the existence of a significant difference between the silvopastoral systems (p = 0.0042). The silvopastoral system with cypress presents a higher mechanical resistance of 2.62 ± 0.98 kg/cm^2^. These results are lower than those reported by Noguera and Vélez (2011), who showed a resistance of 30.59 kg/cm^2^ in an extensive grassland grazing system. This difference could be associated with the animal load of each silvopastoral system (Medina, 2016).

**Figure 2:**
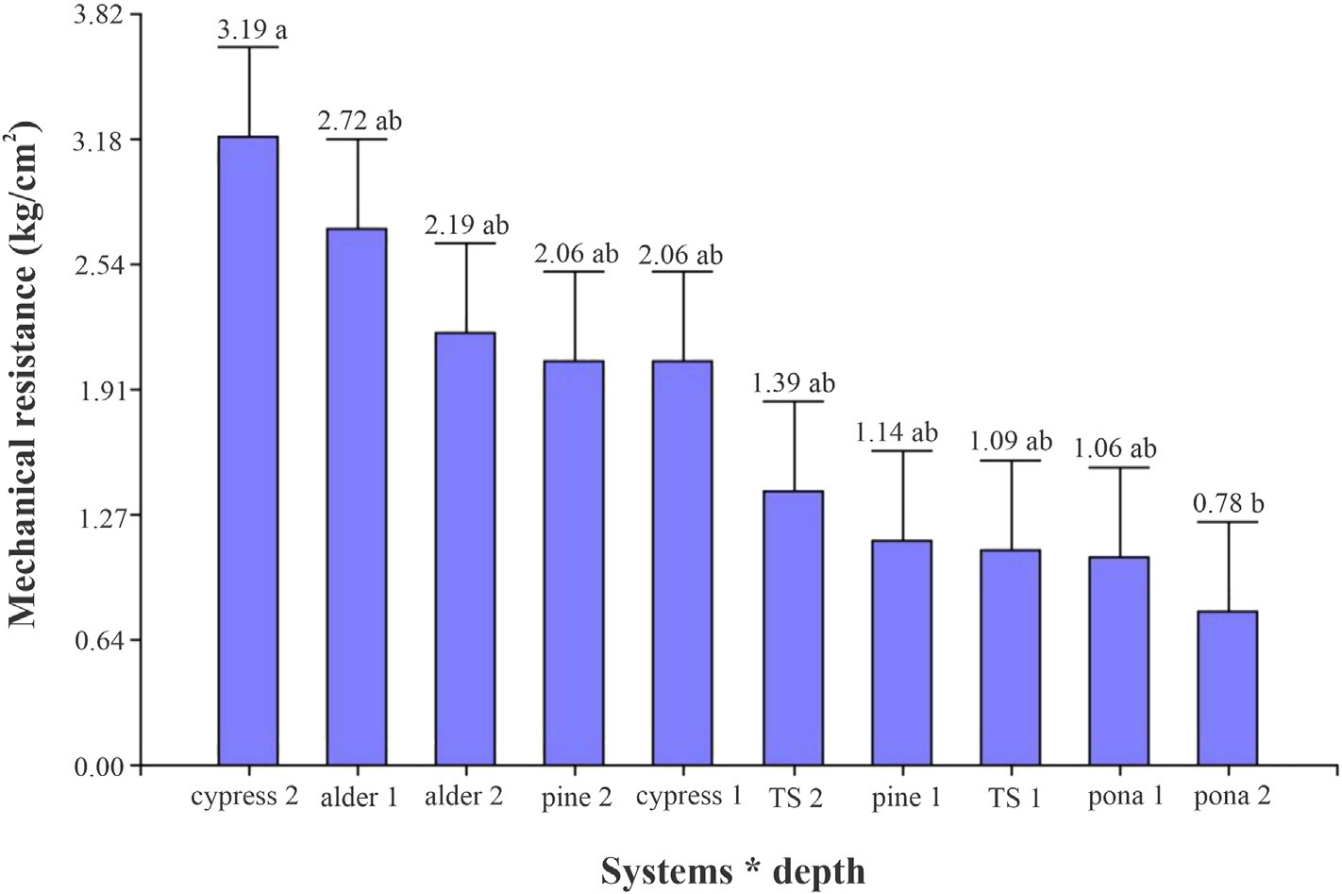
Evaluation of the mechanical resistance of the soil at different depths: 1 (0–15 cm) and 2 (15–30 cm).

Regarding the interaction of the system with depth, the highest resistance was obtained from 15 to 30 cm in the system with cypress (3.19 kg/cm^2^), and the lowest resistance was obtained in the system with pona at a depth of 15–30 cm with 0.78 kg/cm^2^ (Figure 2). Romero et al. (2021) showed similar behaviors with respect to depths in a silvopastoral system with *Acacia*, which could be due to the presence of leaf litter produced by the species.

#### 3.2.2 Bulk density

Bulk density for the different systems evaluated did not show significant differences (p = 0.6482), indicating that this variable does not contribute to determining the effects that the silvopastoral systems being studied may have on the physical properties of the soil. Similar results were reported by Novillo et al. (2018), who evaluated land use systems at different depths from 0.10 m to 0.6 m, where density did not show significant differences.

In the evaluations at two depths, the bulk density showed significant differences (p = 0.0001), suggesting that this variable increased as the evaluation depth increased. The highest bulk density was reported at a depth of 15–30 cm (1.14 ± 0.18), and the lowest was reported at 0–15 cm (0.84 ± 0.11). These results are in agreement with the results reported by Leyva et al. (2018), who, for silvopastoral systems, obtained densities from 1.12 to 1.35 at a depth of 0–30 cm.

For bulk density, the interactions between system and depth showed no significant differences (p = 0.5507); the silvopastoral system with alder evaluated at 15–30 cm showed the highest value of 1.12 ± 0.05 g/cm^3^, while the lowest values corresponded to the pine, cypress and treeless systems at a depth of 0–15 cm (Table 3). These low values could be because pine and cypress generate a superficial layer with the fall of leaves forming superficial organic matter (Chavarría et al., 2012), and in the case of the TS, anthropic intervention could be the cause, indicating poor soil conservation in this type of system (Salamanca and Sadeghian, 2005).

**Table 3:** Physical characteristics of the soil based on the interaction between system (S) and depth (P).

Soil physical characteristics			
Interaction (S × P) (cm)	Density	% Moisture	% Porosity
SPS with alder 0–15	0.84 ± 0.16 b	55.14 ± 19.00 ab	68.41 ± 6.11 ab
SPS with alder 15–30	1.12 ± 0.05 ab	40.87 ± 4.74 ab	57.73 ± 1.93 ab
SPS with pine 0–15	0.79 ± 0.80 b	63.71 ± 14.80 ab	69.99 ± 3.07 ab
SPS with pine 15 – 30	1.26 ± 0.18 a	37.28 ± 8.78 ab	52.53 ± 6.67 b
SPS with cypress 0 – 15	0.79 ± 0.17 b	52.60 ± 23.39 ab	70.14 ± 6.50 a
SPS with cypress 15 – 30	1.21 ± 0.19 a	32.47 ± 10.04 b	54.08 ± 7.26 ab
SPS with pona 0 – 15	0.82 ± 0.07 b	74.54 ± 13.41 a	69.02 ± 2.73 ab
SPS with pona 15 – 30	1.07 ± 0.28 ab	50.37 ± 21.54 ab	59.79 ± 10.51 ab
TS 0 – 15	0.79 ± 0.09 b	69.84 ± 6.73 ab	70.01 ± 3.03 ab
TS 15 – 30	1.01 ± 0.09 ab	56.13 ± 8.55 ab	61.89 ± 6.97 ab

#### 3.2.3 Moisture content

The nature of the soil characterizes the physical properties that influence certain characteristics, such as the moisture percentage (González et al., 2009). This variable reported significant differences (p = 0.0013) at different levels of depth, and the highest moisture percentage was present in the surface layer (0–15 cm), at 0.25 ± 0.17 %, which may be due to the higher production of fine roots (Hernández and Sánchez, 2012).

For this variable, the interaction between system and depth did not show significant differences (p = 0.9155), and the system associated with *Ceroxylon quindiuense* (also known as “pona”) at a depth of 0–15 cm presented the highest percentage of moisture at 74.54 ± 13.41 %. The higher percentage of moisture in this species is because it is a monocotyledonous palm characteristic of montane rainforests (Gómez-Zapata and Salazar-Yepes, 2017).

#### 3.2.4 Porosity

Regarding porosity, there were significant differences at different depths (p = 0.0000), with values higher than 60 % in all systems. The results show a directly proportional relationship between porosity and organic matter content; as the organic matter content increases, the porosity increases (Singh et al., 2018). High porosity values indicate that these soils have adequate aeration and allow adequate plant rooting; porosity values decrease as depth increases, similar to those results obtained by Leyva et al. (2018), who found values of 52.12 (0–20 cm) and 46.85 (20–30 cm) for a silvopastoral system with *Leucaena leucocephala* in strips and *Panicum maximum* grass.

#### 3.2.5 Carbon by depth

Soil carbon stocks showed a significant difference by depth (p = 0.0000), with the highest amounts found in the first 15 cm (91.51 ± 17.95 t/ha); at greater depth, the stored carbon content decreased, presenting a value of 32.26 ± 14.69 t/ha. This result is similar to those reported by Ibrahim et al. (2006), who indicated that this variation depends on the type of soil and the content and decomposition of organic matter of each tree species. Furthermore, this pattern is a very common natural phenomenon in primary forests and silvopastoral systems (Food and Agriculture Organization, 2002).

**Figure 3:**
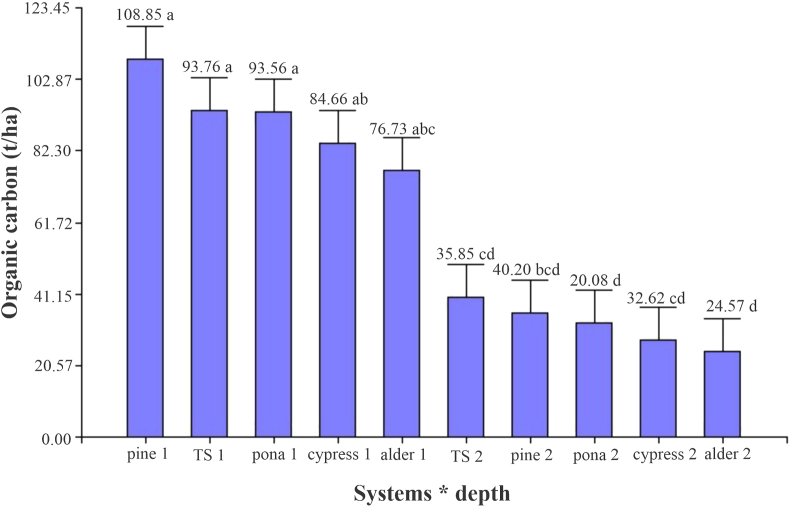
Evaluation of soil carbon in the different systems at different depths: 1 (0–15 cm) and 2 (15–30 cm).

Figure 3 shows a higher carbon accumulation in the system with pine at the 0–15 cm depth, with a value of 108.85 ± 22.55 t/ha, and the lowest value was found for the SPS with alder (24.57 ± 7.89 t/ha) at a depth of 15–30 cm. A similar trend was described by Sokolowska et al. (2020) for three types of land use in Poland: grassland, succession, and forest.

#### 3.2.6 Total organic carbon

In relation to this variable, there were highly significant differences between systems (p = 0.0001). The SPS with pine presented the highest accumulation of total carbon at 149.05 ± 15.27 t/ha, followed by the TS (129.61 ± 8.77 t/ha), and the system with alder (101.30 ± 5.96 t/ha) presented the lowest accumulation. These results are higher than those reported by Alegre et al. (2002), who described a carbon accumulation of 72.5 t/ha for Caucho silvopastoral systems in Pucallpa, Peru, and Lok et al. (2013), who reported carbon in an SPS composed of *Panicum maximum* and *Leucaena leucocephala* in Cuba with a value of 65.3 t/ha. Similarly, Oliva et al. (2017) reported a carbon content in an SPS with pine of 81.24 t/ha in Molinopampa, Peru. This result may be because pine plantations tend to sequester organic carbon according to their plantation age, as mentioned by Reyes et al. (2019), who found higher carbon concentrations for older pine plantations. In addition, vegetation cover behaves in different ways; the organic matter from pine contains lignin (Bernabé-Santiago et al., 2013), causing the total soil organic carbon to stabilize for years (López et al., 2017).

## 4 Conclusions

The chemical characteristics of the soil were similar in the silvopastoral systems and superior to the system without trees. The silvopastoral system with pine had a higher organic matter content, and the silvopastoral system with cypress had high phosphorus and potassium contents. The distribution of trees and the established forest species had an effect on pH, with lower levels in silvopastoral systems with pona and pine. Mechanical resistance in silvopastoral systems is directly related to depth. The highest total carbon sequestration was in the silvopastoral system with pine; for all the evaluated systems, the highest organic matter content was found in the first 15 cm. The use of silvopastoral systems in livestock production is an alternative for the recovery of soils, as it has a positive influence on the physical-chemical quality of the soils.

## Declarations

### Author contribution statement

Héctor V. Vásquez: Conceived and designed the experiments; Performed the experiments.

Leandro Valqui: Analyzed and interpreted the data.

Leidy G. Bobadilla; Jorge L. Maicelo: Conceived and designed the experiments; Wrote the paper.

Carlos I. Arbizu: Analyzed and interpreted the data.

Julio C. Alegre: Contributed reagents, materials, analysis tools or data.

### Funding statement

Héctor V. Vásquez was supported by 10.13039/501100011003Instituto Nacional de Innovación Agraria (INIA).

### Data availability statement

Data included in article/supplementary material/referenced in article.

### Declaration of interests statement

The authors declare no conflict of interest.

### Additional information

No additional information is available for this paper.
